# The changing association between socioeconomic circumstances and the incidence of colorectal cancer: a population-based study

**DOI:** 10.1038/bjc.2011.149

**Published:** 2011-05-10

**Authors:** R Oliphant, D H Brewster, D S Morrison

**Affiliations:** 1 West of Scotland Cancer Surveillance Unit, Department of Public Health, 1 Lilybank Gardens, University of Glasgow, Glasgow G12 8RZ, UK; 2 Scottish Cancer Registry, Information Services Division, NHS National Services Scotland, 1 South Gyle Crescent, Edinburgh EH12 9EB, UK

**Keywords:** colorectal cancer, incidence, socioeconomic circumstance, deprivation

## Abstract

**Background:**

There is emerging evidence to suggest that the association between socioeconomic circumstances and colorectal cancer incidence has changed over recent decades.

**Methods:**

We conducted a descriptive population-based study to describe the relationship between socioeconomic circumstances and the incidence of colorectal cancer in a pre-screened population. Incident cases of colorectal cancer from the West of Scotland were identified from the Scottish Cancer Registry and European age-standardised incidence rates (EASR) were calculated. Socioeconomic circumstances were measured using the area-based Scottish Index of Multiple Deprivation (SIMD).

**Results:**

In total, 14 051 incident cases of colorectal cancer were recorded from 1999 to 2007. Incidence of colorectal cancer was associated with increased deprivation in men but not among women; an association that became evident from 2005 onwards. From 2005 to 2007, the deprivation gap in incidence among men was 13.3 per 100 000 (95% confidence interval 3.2–23.4), with rates 19.5% lower among the least deprived compared with the most deprived. This deprivation gap now accounts for an estimated 75 excess cases per year of male colorectal cancer in the West of Scotland.

**Conclusion:**

Deprivation was associated with higher incidence rates of male, but not female, colorectal cancer before the implementation of a national bowel screening programme.

Colorectal cancer is the fourth most common cancer diagnosed worldwide and is the second most common cause of cancer death in the western Europe after lung cancer (Cunningham *et al*, 2010; Information and Statistics Division, 2010). Since the early 1970s, its incidence has increased in Scotland, especially among men (Gray *et al*, 2002; Information and Statistics Division, 2010), such that the country has the highest incidence of colorectal cancer in both men and women in the United Kingdom (Westlake, 2008). There is emerging evidence that socioeconomic patterns in colorectal cancer incidence have changed direction over the recent decades. In the 1980s, affluence was associated with an increased risk of colorectal cancer (Sharp *et al*, 1993), especially colon cancer (Faggiano *et al*, 1997), but by the end of the decade no association was apparent (Pollock and Vickers, 1997; Harris *et al*, 1998; McLaren and Bain, 1998). By the early 1990s, despite the continued absence of an overall socioeconomic pattern, a small excess of rectal cancers, in men only, was reported in more deprived groups in England and Wales (Quinn *et al*, 2001), although not in all areas (Lawrence *et al*, 2002). Over a similar period, a weak association between colorectal cancer and deprivation was reported in Northern Ireland in both sexes that was more pronounced in men (Kee *et al*, 1996). In the decade to the year 2004 in England, a clearer excess of colorectal cancers was reported in more deprived men, only. Its magnitude changed little between the late 1990s and the early 2000s (Forman, 2010). One difficulty in comparing socioeconomic analyses of colorectal cancer incidence has been that the successive reports have used different methodologies to classify deprivation. Given the evolving changes in the socioeconomic patterns for colorectal cancer and the potential artifacts that national bowel screening programmes may have on subsequent incidence estimates, further detailed analysis, using the most recent cancer registry data, is required.

Many of the established modifiable risk factors for colorectal cancer have changed over time, and some vary by socioeconomic status. There is convincing evidence that consumption of red and processed meat, alcoholic drinks, obesity and smoking comprise the main modifiable risk factors for colorectal cancers (Giovannucci, 2001; American Institute for Cancer Research, 2007). Some of these risk factors have been more common in more deprived populations within the UK. In Scotland, poorer dietary quality and a higher prevalence of smoking have been clearly associated with deprivation over the last 20 years (Dong and Erens, 1997; Bromley *et al*, 2010). However, the relationships among obesity, alcohol consumption and socioeconomic status are less clear. For example, raised waist–hip ratio is only associated with deprivation in women (Dong and Erens, 1997; Hirani, 2009; Bromley *et al*, 2010). Men and women in higher income groups are more likely to be hazardous drinkers, but harmful drinking is more common among the poorest households (Dong and Erens, 1997; Bromley *et al*, 2010). Thus, the relationship between established risk factors and observed colorectal cancer incidence is not a simple one. Other factors, such as earlier detection of premalignant polyps in more affluent populations (Wardle *et al*, 2005; Atkin *et al*, 2010) may contribute to changing socioeconomic associations with incidence.

Our aim was to describe the relationship between colorectal cancer incidence and socioeconomic circumstances, using consistent methodological approaches, both over a period in which changing patterns have been reported and in the most recent period for which cancer registry data were available. Our study period ends immediately before the complete introduction of the Scottish Bowel Screening Programme. We studied a geographical area with wide variations in socioeconomic circumstances and a large population of deprived individuals.

## Materials and methods

The West of Scotland has a population of 2.4 million and comprises approximately half of the Scottish population. Incident cases of colorectal cancer diagnosed from 1 January 1999 to 31 December 2007 in this region were extracted from the Scottish Cancer Registry using the International Classification of Diseases version 10 (ICD-10) codes for colorectal cancer (C18–C20). Appendiceal (C18.1) cancers were excluded because they are pathologically and clinically different from other colorectal cancers.

The Scottish Cancer Registry (SMR 06) records all incident cancers in Scotland, and since 1997 registration has been centralised at the Information and Services Division (ISD) of the National Health Service National Services Scotland. Patient data include date of birth, sex, postcode, date and details of diagnosis. At the time of extraction, incident data were available to the end of 2007.

Socioeconomic circumstances were measured using the Scottish Index of Multiple Deprivation 2006 (SIMD) (Scottish Government, 2010). It provides an area-based measure of socioeconomic circumstance, based on the postcode of residence. There are 6505 geographical small areas or data zones across Scotland; each containing approximately 750 people. The SIMD score provides a relative ranking of these 6505 areas from the most to the least deprived, based on detailed information on seven key subject areas including: (1) income and benefits (2) employment in working age population (3) health and healthcare utilisation (4) educational attainment, skills and training (5) access to services and transport (6) recorded crime rates and (7) housing quality and overcrowding. The score generated for each key subject area (weighted towards income, employment and education) is ultimately combined to create an overall SIMD score for each data zone. Overall, SIMD 2006 scores are presented as quintiles, with 1 representing the least deprived and 5 representing the most deprived; each representing 20% of the Scottish population. Individual SIMD scores can then be assigned to the population, based on the postcode of residence. Scottish Index of Multiple Deprivation-specific yearly population estimates by age and sex for the West of Scotland were obtained from ISD, from 1999 to 2007.

### Statistical methods

Age-standardised incidence rates were calculated by direct standardisation to the European Standard Population (Waterhouse *et al*, 1976) to control for differences in the age structure of the population between deprivation quintiles and over time. All rates are presented as European age-standardised rates (EASR per 100 000), with 95% confidence intervals (CI) where appropriate. The rates presented by deprivation quintile have been calculated as 3-year averages to reduce the effects of year-to-year random variation due to small numbers. The test for trend was calculated from the weighted ordinary least squares linear regression with *P*-value <0.05 regarded as statistically significant. The deprivation gap in incidence is the absolute rate difference in EASR per 100 000 and is also presented as the percentage difference between the most and least deprived, derived from the ordinary least squares linear regression. The EASR ratio was calculated by dividing the EASR of each deprivation quintile with the corresponding EASR of the least-deprived quintile. A crude estimation of the number of excess cases for each deprivation quintile was calculated as the difference between the original number of incident cases and the estimated number of incident cases had each deprivation group the EASR of the least deprived quintile. This was only presented when the corresponding regression analysis showed a significant trend across deprivation quintiles. All data handling and analysis was carried out using Stata v11 IC (Statacorp, College Station, TX, USA).

## Results

There were 14 051 incident cases of colorectal cancer in the West of Scotland from 1 January 1999 to 31 December 2007, of which 53.6% were male. The mean age at diagnosis was higher in women compared with men (72.3 *vs* 70 years, respectively, *P*<0.001, *t*-test). A comparison of the socioeconomic characteristics of colorectal cancer patients with the general West of Scotland population is provided in Table 1.

**Figure 1:**
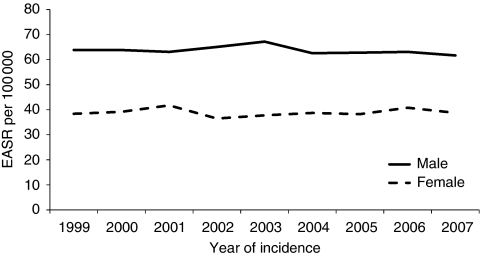
European age-standardised colorectal cancer incidence in the West of Scotland, by sex, 1999–2007.

The overall age-standardised incidence of colorectal cancer in 2007 was 61.7 per 100 000 (95% CI 57.5–65.8) in men and 38.8 per 100 000 (95% CI 35.8–41.7) in women. There was no change in incidence rates in either men or women over time (Figure 1).

**Figure 2:**
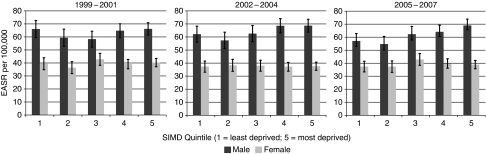
European age-standardised colorectal cancer incidence in the West of Scotland, by sex and Scottish Index of Multiple Deprivation (SIMD) quintile, 1999–2007.

There was no consistent relationship between colorectal cancer incidence and socioeconomic circumstances in either 1999–2001 or 2002–04, although incidence rates were highest among men in the most deprived groups (SIMD 4 and 5) in 2002–2004 (Figure 2). During the latest period, 2005–2007, an association between increasing levels of deprivation and male colorectal cancer incidence became clearer (*P*=0.025, trend) (Figure 2), mainly due to the reduction in rates among the most affluent. Incidence rates fell between 1999 and 2007 among men in the most affluent areas only (*P*=0.048, trend), but we found no other trend among other deprivation groups in either males or females. From 2005 to 2007, the incidence among the most deprived males was 69.0 per 100 000 (95% CI 64.1–73.8) compared with 57.1 per 100 000 (95% CI 51.4–62.8) for the least deprived (Table 2), giving the deprivation gap as an absolute rate difference of 13.3 per 100 000 (95% CI 3.2–23.4). Incidence rates among the most affluent males were 19.5% lower than those in the most deprived during the latest period (2005–2007). No similar association was seen among women (Table 3). An estimated 75 fewer cases of male colorectal cancer per year would be diagnosed in the West of Scotland if all socioeconomic deprivation quintiles had the rate of the least deprived from 2005 to 2007.

## Discussion

Deprivation has become associated with higher incidence rates of colorectal cancer in men, but there is no clear association in women. The rise in incidence of colorectal cancer that occurred from the 1970s appears to have levelled-off, but a deprivation gap in men has emerged as a result of a relative reduction in incidence among the least deprived. Colorectal cancer incidence rates in males were 19.5% lower among the least deprived compared with the most deprived during the latest period (2005–2007). This emerging deprivation gap accounts for an estimated excess of 226 cases of male colorectal cancer in the West of Scotland during this period, approximately 9% of all male cases. Although this deprivation gap is smaller in magnitude to that reported for lung cancer, for example, the public health implications of this changing association before the implementation of a national bowel screening programme are compelling. Future incidence trends should be closely monitored, as further socioeconomic inequalities in colorectal cancer are likely to become increasingly evident.

Our findings are consistent with earlier reports of an emerging association between male, but not female, colorectal cancer and deprivation in the United Kingdom (Kee *et al*, 1996; Quinn *et al*, 2001; Forman, 2010) using a variety of socioeconomic indices. The emerging deprivation gap in male colorectal cancer incidence in this series is larger than reported for England from 2000 to 2004 (Forman, 2010). This could suggest that the West of Scotland population has a wider background spectrum of deprivation or that the deprivation gap is widening on a national level over time. From 1970 onwards in Europe, the majority of evidence indicates that socioeconomic deprivation is associated with a lower risk of colorectal cancer (Faggiano *et al*, 1994; van Loon *et al*, 1995; Aarts *et al*, 2010), whereas over the same period in North America and Canada, the incidence is higher among the more deprived groups (Gorey and Vena, 1995; Mackillop *et al*, 2000). This highlights the dynamic nature of the relationship between socioeconomic circumstance and colorectal cancer incidence in Scotland where previously incidence rates were associated with affluence in both sexes (Sharp *et al*, 1993). This international variation is likely to be a combination of varying exposures to modifiable lifestyle-related risk factors and access to large bowel investigations and colorectal cancer screening. However, the exact mechanism remains unclear.

The majority of colorectal cancers are sporadic and related to lifestyle factors, including diet (high in red and processed meat and alcohol (in men) and low in dietary fibre, garlic, milk and calcium) (American Institute for Cancer Research, 2007; Chan and Giovannucci, 2010; Kirkegaard *et al*, 2010), smoking (Giovannucci, 2001; Cleary *et al*, 2010), obesity, and low levels of physical activity (Knekt *et al*, 1998; American Institute for Cancer Research, 2007; Chan and Giovannucci, 2010; Kirkegaard *et al*, 2010). In Scotland, over the last few decades, smoking, obesity and poor diet (higher consumption of non-diet drinks, crisps, savoury snacks, chips and meat products; lower consumption of fruit and vegetables) have been more prevalent among the most deprived (Dong and Erens, 1997; Bromley *et al*, 2010). In addition, the relationship between known risk factors and socioeconomic status in many cases is nonlinear. For example, despite an overall reduction in the prevalence of smokers in Scotland over the past 20 years, smoking is not only more common among the most deprived but also smokers from deprived areas tend to smoke more heavily than those from affluent areas (Dong and Erens, 1997; Bromley *et al*, 2010).

The underlying reason for the emergence of an association between colorectal cancer incidence and deprivation in men, but not in women, remains uncertain. Some differences in risk factors between sexes may partially explain the observed differential in socioeconomically patterned incidence, such as the observed excess of binge drinking in men and lower levels of physical activity in women (Bromley *et al*, 2010). Therefore, the emerging socioeconomic gradient in male colorectal cancer incidence may reflect an overall reduction in exposure to modifiable risk factors among the least deprived, rather than pointing to one specific explanatory socially determined colorectal cancer risk factor. For example, smoking rates in Europe have reduced to a greater extent among the most educated men compared with those with lower educational attainment over recent decades (Giskes *et al*, 2005). In addition, in Scotland from 1986 to 1995, fruit and vegetable intake increased among the least, but not most, deprived groups leading to a widening social gradient (Wrieden *et al*, 2004). However, the exact mechanism of risk factor interaction remains unclear, as the lack of a socioeconomic gradient among women cannot be explained by pooling of risk factors among the most deprived. It seems reasonable to suggest, however, that many of the risk factors for cardiovascular diseases, diabetes mellitus and several cancers, including those of the colorectum, are shared and that more effective health improvement initiatives – particularly those directed at reducing socioeconomic inequalities – are required.

This widening socioeconomic disparity among males is noteworthy, as it has emerged before the implementation of the Scottish Bowel Screening Programme launched in 2007, with a staggered introduction over a 3-year period. Results from pilot studies and organised screening programmes using fecal occult blood test (FOBt) in the UK (Alexander and Weller, 2003; Von Wagner *et al*, 2011) and Europe (Frederiksen *et al*, 2010) have reported poorer overall uptake among deprived groups and in males. If the socioeconomic uptake differentials reported from pilot screening trials in Scotland continue within the nationally coordinated programme, an initial attenuation of the incidence inequalities reported here can be expected because of detection of prevalent cases in screening participants. However, should screening lead to a long-term reduction in incidence and mortality among participants, a widening deprivation gap may develop leading to further socioeconomic health inequality. Therefore, future incident trends and screening participation require careful monitoring. In addition to formal screening programmes, there may also be socioeconomic differences in self-presentation, referral and treatment patterns for pre-malignant polyps of the large bowel that may prevent subsequent cancerous transformation.

A particular strength of this study is the incidence data, obtained from the Scottish Cancer Registry, are of high quality, accurate and have high levels of case ascertainment (Brewster *et al*, 1995, 1997; Harris *et al*, 1998). The use of data from a single region and registry also reduces the potential confounding effect of variations in registration or coding practices that could be encountered if using data from multiple registries.

No universally accepted definition of deprivation exists, but it reflects the end of a spectrum of material and social disadvantage with relative to others in a defined population. As an area-based measure of deprivation, SIMD is now the index of choice of the Scottish Government and the ISD, Scotland. It can be estimated by using a standard methodology, and is readily available for the entire population for which postcode of residence is known. However, as with all area-based measures of socioeconomic circumstances, it does make the assumption that all people living in the same geographical area assume the same level of deprivation, and there is no allowance for non-deprived people living in an area of deprivation. Individual-level data on socioeconomic status such as income or educational attainment would reduce such ecological fallacy, however, these are not routinely collected in the SMR 06. Scottish Index of Multiple Deprivation is also not a measure of affluence, and therefore, all that can be said about the highest ranked areas is that there is less deprivation. It is Scotland-specific and therefore cannot be used to directly compare other regions of the United Kingdom, although the English Index of Multiple Deprivation is similar.

The West of Scotland has a proportionally larger deprived and smaller affluent population than Scotland as a whole, as measured by the SIMD 2006 score (Table 1). However, as Scotland-weighted SIMD 2006 scores and age-, sex- and SIMD 2006-specific yearly population estimates were used, this should not influence the results presented. In addition, the proportion of the West of Scotland population within each deprivation group did not change significantly over the 9-year period, that is, from 1999 to 2007, therefore, any changes in the rates presented are unlikely to have been influenced by variation of the socioeconomic make-up of the population.

Within the study population, one hospital took part in a multicentre randomised screening trial of once-only sigmoidoscopy (Atkin *et al*, 2010) from November 1994 to March 1999. In total, 2986 persons between the ages of 55 and 64 years underwent a screening sigmoidoscopy during this period. The trial hospital had a highly deprived catchment area where almost 45% of the population was within the most deprived quintile for Scotland. Even allowing for an initial increase in incidence, followed by a later reduction in rates among trial participants, as it could be expected after the implementation of such a screening programme, we feel that this had little bearing on our overall findings. In addition, one NHS Health Board within the study area started using FOBt screening in September 2007 as part of the Scottish Bowel Screening Programme. Owing to the short period of time, this screening programme had been running within our study period, and with results from the pilot screening study showing low uptake rates among the most deprived males (Alexander and Weller, 2003), it is unlikely that this significantly altered our results.

Further areas for study should focus on the reasons for the growing deprivation gap in incidence in men because of a reduction among the least deprived to identify potential aspects of modifiable risk, behaviour or healthcare provision with which this inequity could be addressed, leading to an overall reduction in colorectal cancer. Bowel cancer screening participation rates should be closely monitored among deprived men to enable effective targeting of screening strategies to avoid compounding this health inequality.

## Conclusion

Deprivation has become associated with higher rates of male, but not female, colorectal cancer with a widening deprivation gap in male incidence emerging in the West of Scotland because of a reduction among the least deprived. This may reflect beneficial changes in a number of modifiable risk factors for colorectal cancer among the more affluent populations. This deprivation gap requires careful observation to ensure any effect of the Scottish Bowel Screening Programme on colorectal cancer incidence does not lead to further health inequality among the most deprived. Further investigation to find out reason reasons for this socioeconomic differential is also required.

## Figures and Tables

**Table 1:** Summary patient demographics (1999–2007) and population characteristics (2006) for the West of Scotland

	**Incident colorectal cancer cases 1999–2007 (%)**			**Population estimate 2006 (%)**		
**Deprivation group**	**Men**	**Women**	**All**	**Men**	**Women**	**All**
Least deprived	1182 (15.7)	948 (14.6)	2130 (15.2)	194 968 (16.9)	205 351 (16.4)	400 319 (16.7)
2	964 (12.8)	862 (13.2)	1826 (13)	172 208 (15)	182 503 (14.6)	354 711 (14.8)
3	1179 (15.6)	1103 (16.9)	2282 (16.2)	191 263 (16.6)	203 903 (16.3)	395 166 (16.5)
4	1850 (24.6)	1602 (24.6)	3452 (24.6)	255 930 (22.2)	283 297 (22.6)	539 227 (22.4)
Most deprived	2361 (31.3)	2000 (30.7)	4361 (31)	336 225 (29.2)	376 452 (30.1)	712 677 (29.7)
Total	7537 (53.6)	6515 (46.4)	14 051	1 150 594 (47.9)	1 251 506 (52.1)	2 402 100

**Table 2:** Male colorectal cancer incidence by SIMD, West of Scotland, 1999–2007

**Deprivation group**	**Number of cases**	**European age-standardised rate (EASR) per 100 000**	**95% Confidence intervals**	**EASR ratio**	**Estimated excess cases**
*1999–2001*					
Least deprived	386	66.1	(59.5, 72.8)	1	
2	309	59.4	(52.8, 66.1)	0.9	No
3	359	58.3	(52.2, 64.4)	0.9	significant
4	595	64.8	(59.5, 70.1)	1	difference
Most deprived	774	66.2	(61.5, 71.0)	1	
Overall	2424	63.6	(61.0, 66.1)		
*P*-value for trend		0.707			
					
*2002–2004*					
Least deprived	399	62.2	(56.1, 68.4)	1	
2	322	57.4	(51.1, 63.7)	0.9	No
3	401	62.6	(56.4, 68.8)	1	significant
4	637	68.6	(63.1, 74.0)	1.1	difference
Most deprived	785	68.7	(63.8, 73.6)	1.1	
Overall	2544	64.9	(62.4, 67.5)		
*P*-value for trend		0.106			
					
*2005–2007*					
Least deprived	397	57.1	(51.4, 62.8)	1	0
2	333	54.7	(48.8, 60.6)	0.9	−15
3	419	62.3	(56.3, 68.3)	1.1	35
4	617	64.1	(59.0, 69.3)	1.1	67
Most deprived	802	69.0	(64.1, 73.8)	1.2	138
Overall	2568	62.5	(60.1, 65.0)		226
*P*-value for trend		0.025			

**Table 3:** Female colorectal cancer incidence by SIMD, West of Scotland, 1999–2007

**Deprivation group**	**Number of cases**	**European age-standardised rate (EASR) per 100 000**	**95% Confidence intervals**	**EASR ratio**	**Estimated excess cases**
*1999–2001*					
Least deprived	301	39.4	(34.8, 43.9)	1	
2	261	36.4	(31.8, 41.0)	0.9	No
3	373	42.8	(38.2, 47.3)	1.1	significant
4	537	39.1	(35.5, 42.7)	1	difference
Most deprived	711	40.3	(37.1, 43.5)	1	
Overall	2183	39.8	(38.0, 41.5)		
*P*-value for trend		0.613			
					
*2002–2004*					
Least deprived	314	37.3	(33.0, 41.5)	1	
2	297	38.3	(33.7, 42.9)	1	No
3	341	38.0	(33.8, 42.3)	1	significant
4	517	37.2	(33.8, 40.7)	1	difference
Most deprived	644	37.6	(34.5, 40.7)	1	
Overall	2113	37.7	(36.0, 39.4)		
*P*-value for trend		0.785			
					
*2005–2007*					
Least deprived	333	37.4	(33.3, 41.6)	1	
2	304	37.4	(33.0, 41.8)	1	No
3	389	42.9	(38.4, 47.4)	1.1	significant
4	548	39.8	(36.2, 43.4)	1.1	difference
Most deprived	645	38.9	(35.7, 42.1)	1	
Overall	2219	39.3	(37.5, 41.0)		
*P*-value for trend		0.532			
